# [Expression of Concern] Adenovirus-mediated truncated Bid overexpression induced by the Cre/LoxP system promotes the cell apoptosis of CD133^+^ ovarian cancer stem cells

**DOI:** 10.3892/or.2026.9121

**Published:** 2026-04-15

**Authors:** Qifang Long, Ru Yang, Weixian Lu, Weipei Zhu, Jundong Zhou, Cui Zheng, Dongmei Zhou, Ling Yu, Jinchang Wu

Oncol Rep 37: 155–162, 2017; DOI: 10.3892/or.2016.5263

Following the publication of the above paper, it was drawn to the Editor's attention by a concerned reader that a section of the western blot image for β-actin in Fig. 1B on p. 157 had also been used for the control β-actin blots in Fig. 3C on p. 159, despite these data having come from different sources (Fig. 1: CD133^-^ ovarian cancer cells compared with Fig. 3: CD133^+^ ovarian cancer stem cells infected with recombinant adenovirus). In addition, upon performing an independent analysis of the data in this paper in the Editorial Office, it also came to light that, regarding the TUNEL assay experiments shown in Figs. 2 and 4, the data shown for panel ‘1’ in Fig. 2D was strikingly similar to that shown for the ‘Blank’ panel in Fig. 4C, even though the experimental conditions in these figures were reported to be different.

The authors have been contacted by the Editorial Office to offer an explanation for this apparent duplication of data within these figures, and we are waiting their response. Owing to the fact that the Editorial Office has been made aware of potential issues surrounding the scientific integrity of this paper, we are issuing an Expression of Concern to notify readers of these potential problems while the Editorial Office continues to investigate this matter further.

